# Vegetative Propagation of *Hoya imperialis* and *Hoya coronaria* by Stem Cutting and Micropropagation

**DOI:** 10.21315/tlsr2021.32.3.1

**Published:** 2021-09-30

**Authors:** Siti Madihah Mohd Don, Nur Maziyyah Abdul Hamid, Hussein Taha, Rahayu Sukmaria Sukri, Faizah Metali

**Affiliations:** 1 Environmental and Life Sciences Programme, Faculty of Science, Universiti Brunei Darussalam, Jalan Tungku Link, BE1410, Brunei Darussalam; 2 Institute for Biodiversity and Environmental Research, Universiti Brunei Darussalam, Jalan Tungku Link, BE1410, Brunei Darussalam

**Keywords:** Auxins, Callus Induction, Cytokinins, *Hoya coronaria*, *Hoya imperialis*, Stem Cutting

## Abstract

*Hoya imperialis* (*H. imperialis*) and *H. coronaria* (Apocynaceae) are known to have ornamental value due to their beautiful flowers; however, the feasibility of propagating these plants have not been reported despite the wild populations in Brunei Darussalam being highly threatened due to habitat loss and overcollection. Thus, the present study aimed to conduct a preliminary study of the feasibility of two alternative propagation methods, stem cutting and micropropagation, as a potential approach for their *ex situ* conservation. *Hoya* stem cuttings were treated with either indole-3-butyric acid (IBA) or 1-naphthaleneacetic acid (NAA) (0–2000 mg/L), and then propagated onto a mixture of peat moss and perlite. For micropropagation, *Hoya* leaf explants were cultured onto Murashige and Skoog (MS) agar media that were supplemented with IBA and/or kinetin (KN) (0–10.0 mg/L). This present study shows that both *Hoya* species were successfully propagated by stem cutting even without hormone treatment. However, interestingly, in *H. imperialis*, when compared with control, the mean number of new leaves (6.3 ± 1.0) and the mean relative growth rate (RGR) based on stem diameter (0.004 ± 0.0007 cm cm^−1^ day^−1^) significantly increased when treated with 500 mg/L NAA and 2000 mg/L IBA, respectively. Meanwhile, in *H. coronaria*, significantly higher mean number of roots was achieved by treating with 1000 mg/L NAA (16.6 ± 1.4) or 2000 mg/L IBA (17.5 ± 2.7) compared with control. For micropropagation, callus induction was not promising and could only be observed at specific concentrations of both IBA and KN, with *H. imperialis* appearing to be more responsive towards these hormones in comparison to *H. coronaria*. The present study showed that stem cutting appeared more feasible in propagating both *Hoya* species.

**Highlights**

- Vegetative propagation of *Hoya imperialis* (*H. imperialis*) *and H. coronaria* was investigated.
- Positive shoot and root regeneration in stem cuttings using IBA and NAA.
- Positive callus induction of leaf explants in response to IBA and KN.
- Stem cutting was a reliable method for mass propagation of *Hoya* species.

## INTRODUCTION

The genus *Hoya* from the Apocynaceae family is a species-rich genus comprising mainly epiphytes and are geographically distributed in the Indomalesian-Australian region (Kleijn & van Donkelaar 2001). It is one of the most diverse plant genera in the tropical and subtropical regions, such as in South Africa, South America, Southeast Asia and New Guinea (Kloppenburg & Wayman 1992; Rodda *et al*. 2013). Borneo comprises a centre of diversity for *Hoya*, with 71 *Hoya* species and one subspecies recently listed (Rodda 2017). The full extent of *Hoya* diversity in Borneo remains difficult to determine (Choy 2015), mainly due to the remoteness and underexploration of rainforests within the island (Lin *et al*. 2014). For Brunei Darussalam in Northwest Borneo, a preliminary checklist showed this small region of Borneo has 27 *Hoya* species that includes three undescribed taxa (Rodda 2014).

Most *Hoya* species are popularly cultivated for their ornamental values due to their aromatic and beautiful flowers (Lamb & Rodda 2016; Bermuli *et al*. 2019). *Hoya* plants have been introduced as exotic ornamental plants in Europe and the U.S. since 1970s (Bermuli *et al*. 2019) and their popularity has also spread to Australia (Rahayu 2012). This can be seen by the increasing number of webpages, associations and societies that are dedicated to the horticulture, international trades and plant exchanges of *Hoya* in the recent years (Hodgkiss 1997; Rahayu 2018). *Hoya* plants are also often exploited for ethnomedicinal purposes (Heyne 1979; Ambasta & Wickens 1988) and many indigenous tribes use them as medicines and traditional treatments (Zachos 1998). For example, *H. multiflora* was reported to possess a drug compound that could treat several diseases including asthma (Heyne 1979), intestinal inflammations, abdominal pain (Ambasta & Wickens 1988) and arthritis-rheumatic disease (Burkill 2002). *H. lacunosa* and *H. latifolia* were also reported to have the potential to be used as bio-insecticide that could control the growth of pre-adult mosquitoes, *Aedes aegypti* and *Culex quinquefasciatus* which are known vectors of several viruses (Cahyadi 2005; Kusumawati 2005; Mukharam 2005; Rustandi 2005).

The popularity of *Hoya* as ornamental and medicinal plants has increased the pressure to harvest more of these species from the wild, resulting in declining *Hoya* populations in the native forests (Bermuli *et al*. 2019). Deforestation and land-use changes due to land and agricultural developments further contribute to the declining populations of *Hoya* since forest tree trunks act as substrates for climbing *Hoya* species (Rodda & Ang 2012). Another threat is the high demand for *Hoya* in the trade market and horticultural industry. It is therefore important to conserve these *Hoya* plants, and one of the many ways to conserve these valuable plants is via *ex situ* conservation through vegetative propagation methods such as stem cutting or micropropagation.

In this current study, *H. imperialis* and *H. coronaria* were chosen as the study species due to their ornamental values with immense medicinal potentials. These two *Hoya* species are not well studied in Brunei Darussalam and their conservation status is unknown (IUCN 2020). Within Brunei Darussalam, they can be found in a unique tropical heath forest in Tutong District (Din *et al*. 2015) but this area is highly prone to residential and road development which may endanger the wild populations. *H. coronaria* has been considered to be extinct in Singapore (Chong *et al*. 2009), while *H. imperialis* has been listed as vulnerable and endangered in the Philippines (Department of Environment and Natural Resource 2017). Consequently, there is a need to propagate them via *ex situ* conservation approaches to minimise the pressure on their natural populations and to promote sustainable supply of raw materials for complete tapping of their medicinal potentials.

Vegetative propagation of *H. imperialis* and *H. coronaria* was investigated via stem cutting and micropropagation and both methods, which are commonly associated with plant hormone supplementation, have not been previously reported for these two unique plants. Nevertheless, successful plant propagation via stem cutting using exogenous plant hormones had been reported for several local plants in Brunei (Kondo 1989; Bujang 1992; Jani & Hachinohe 1992; Awang Kamis *et al*. 2016; Matali *et al*. 2017). For micropropagation, other *Hoya* species, such as *H. carnosa* (Maraffa *et al*. 1980), *H. kerrii* (Siddique 2013) and *H. wightii* ssp. *palniensis* (Lakshmi *et al*. 2010) have demonstrated the capacity to be successfully propagated in the presence of different concentrations of auxins (indole-3-acetic acid or IAA, indole-3-butyric acid or IBA, 1-naphthaleneacetic acid or NAA or 2,4-dichlorophenoxyacetic acid or 2,4-D) and cytokinins (kinetin or KN and 6-benzyladenine or BA) in the media culture. Therefore, this study aimed to compare stem cutting and micropropagation in propagating *H. imperialis* and *H. coronaria*. IBA and NAA were used in inducing shoot and root productions in *Hoya* stem cuttings, whereas IBA and/or kinetin (KN) were used in inducing callus production. Callus induction is the first step of micropropagation, and once optimal conditions for callus induction are known, further study can be conducted to induce shoots and roots from the callus.

## MATERIALS AND METHODS

### Stem Cutting

#### Sampling sites and stem cutting preparation

Stem cuttings of *H. imperialis* and *H. coronaria* were collected from the heath forest in the white sand area (N 04°44′57.0″, E 114°36′38.3″) and Bukit Beruang (N 04°44′42.4″, E 114°36′09.3″) in Tutong District, Brunei Darussalam. Field identification of stock *H. imperialis* and *H. coronaria* plants was conducted with the assistance of botany staff from the Brunei Forestry Department Herbarium (BRUN). Voucher specimens were collected, and identification was confirmed with the BRUN collection. A detailed description of study sites and an account of their forest structure and species composition are provided by Din *et al*. (2015). Healthy apical stem cuttings were collected in the morning from ten randomly chosen adult stock plants and stored in moistened and sealed plastic bags to minimise water loss. Two-nodal leafy stem cuttings (10 cm–20 cm long and 3 cm–6 cm in diameter for *H. imperialis*; 13 cm–17 cm long and 2.0 cm–4.5 cm in diameter for *H. coronaria*) were prepared with the leaves trimmed to half of their length and with the flowers and buds excised from the cuttings.

#### Propagation of stem cuttings using IBA and NAA

All stem cuttings received a basal quick-dip up to 2 cm for 10 s in distilled water (control) or in liquid hormone, either NAA or IBA (Sigma-Aldrich, USA) at different concentrations (500, 1000 and 2000 mg/L). Each cutting was then initially planted into a pot containing 60 g of 1:1 mixture of autoclaved peat moss (FREE PEAT, Free Peat B. V., Holland) and perlite. Each control or hormonal treatment had 10 pots or replicates. A water mist system was used to provide an overhead 1 min mist at every 5 min interval for 20 weeks. Plastic sheets were also fixed above the pots and the water mist system to maintain high atmospheric humidity and prevent excessive water loss. The mean daily temperature, relative humidity and maximum photosynthetically active radiation (PAR) measured by the Quantum MQ-200 PAR Meter (Apogee Instruments, UK) in the plant house were 25 ± 3°C, 80%–90% and 300 μmol m^−2^ s^−1^, respectively. The pots were randomly re-arranged in the plant house every week. At week 12, successfully propagated cuttings were transferred to medium-sized pots, each containing 800 g of the same substrate. The cuttings were allowed to grow to week 20 before growth assessment was carried out.

#### Assessment of cuttings

All stem cuttings were assessed at week 20. Newly produced leaves were counted per cutting, which were then traced on a blank paper and measured for leaf area (in cm^2^) using the leaf area meter (AM300, ADC BioScientific Limited, TA, UK). All produced roots from each cutting were counted for root number with the longest three of the produced roots from each cutting were measured (in cm) and the mean were recorded to obtain the average root length for both *Hoya* species. In addition, relative growth rate (RGR) in terms of stem diameter (cm) and length (cm) over 20 weeks were calculated following Hunt (1990):


$$\text{RGR}=\frac{{log}_{e}w2-{log}_{e}w1}{t_{2}-t_{1}}$$

where,

*w*2 and *w*1 = the final and initial stem diameter (cm) for RGR stem diameter (cm cm^−1^ day^−1^) or the final and initial stem length (cm) for RGR stem length (cm cm^−1^ day^−1^) and *t*_2_ – *t*_1_ = 140 days (*t*_2_ is week 20 and *t*_1_ is the initial week 0).

#### Field growth of propagated cuttings

After the growth assessment at week 20, representative cuttings (plantlets) were further transferred into larger pots containing 1.3 kg of peat moss supplemented with two granules of slow release fertiliser (NPK 15:15:15) every two weeks. The potted cuttings were moved from the plant house to a plant shade in the field and grown at mean daily temperature of 30 ± 3°C, relative humidity of 60%–75% and maximum PAR of 450 μmol m^−2^ s^−1^. The cuttings were watered to field capacity twice a day and peas stick was used to physically support the growth of these climbers. Field observation was carried out for a period of one year by observing the survival of the plantlets.

### Plant Tissue Culture

#### Preparation of leaf explants

All successfully propagated *H. imperialis* and *H. coronaria* stem cuttings that had grown to adult plants in the stem cutting experiment were used as the stock plants. Young leaves were excised from the stock plants using a sterilised scalpel that had been dipped in 10% Clorox. The leaves were further excised into smaller segments of approximately 0.5 cm × 0.5 cm in size, which were then used as leaf explants. All leaf explants were collected in the morning and stored in moist plastic bags to prevent them from drying. Each leaf explant was gently brushed with a commercial disinfectant (Dettol) using a soft paint brush or toothbrush under running tap water for 10 min.

Inside a sterile laminar flow cabinet, the explant was dipped into 95%– 97% ethanol for 2 s and then rinsed with sterilised distilled water four times. The explant was then soaked in 10% Clorox containing three drops of Tween 20 (Sigma-Aldrich, USA) for 30 min, after which it was rinsed four times with sterilised distilled water. The explant was subsequently dried with sterile tissue papers.

#### Callus induction

Each sterilised leaf explant was cultured onto a sterile Petri dish containing sterilised Murashige and Skoog (MS) basal medium with sucrose and agar (Sigma-Aldrich, USA) at pH 5.6. MS agar plates were supplemented with different concentrations of IBA and KN (0, 0.5, 1, 3, 5 and 10 mg/L). A total of 36 treatments were conducted, including a control treatment (0 mg/L IBA and 0 mg/L KN; Appendices A and B). Each treatment had four MS agar plates or replicates with one explant per plate. The plates were then incubated in a plant growth incubator (LCC-150MP, Daihan Labtech Co., LTD) at a temperature of 23°C–25°C and a cycle of 8-h light and 8-h dark for at least 12 weeks. The images of callus per treatment were captured after 12 weeks and the surface area of callus (area in mm^2^) formed was demarked using the freehand tool ImageJ software that was calibrated via a scale bar embedded within each image (Schneider *et al*. 2012).

### Statistical Analysis

For stem cutting, statistical analysis was carried out for each *Hoya* species (*H. imperialis* and *H. coronaria*) to compare the control treatment (distilled water) with the hormone treatments (IBA or NAA) at three different concentrations (500, 1000 and 2000 mg/L) using a one-way analysis of variance (ANOVA) and Tukey’s honest significant difference (Tukey’s HSD) test at 5% significance level. Assumptions of normality and equal variances were checked using Shapiro Wilk Test and F-Test, respectively and were not violated. For the tissue culture experiment, differences in surface area of callus formed were separately analysed for each *Hoya* species using a Kruskal-Wallis non-parametric test, followed by the Dunn’s pairwise multiple-comparisons test with Bonferroni’s correction at 5% significance level due to the presence of large number of zeros in both dataset and the small number of replications per treatment (*n* = 4). All statistical analyses were conducted using R version 3.4.4 (R Development Core Team 2016).

## RESULTS

### Stem Cuttings

#### Survival percentages

High survivability (100%) of the cuttings was observed in the first 20 weeks for all treatments in both *H. imperialis* and *H. coronaria*. Measurements such as the number of new leaves, leaf area, number of roots, root length and RGR values based on stem length and stem diameter were recorded for both *Hoya* species as described below.

#### Number of new leaves and leaf area

**Figure 1:**
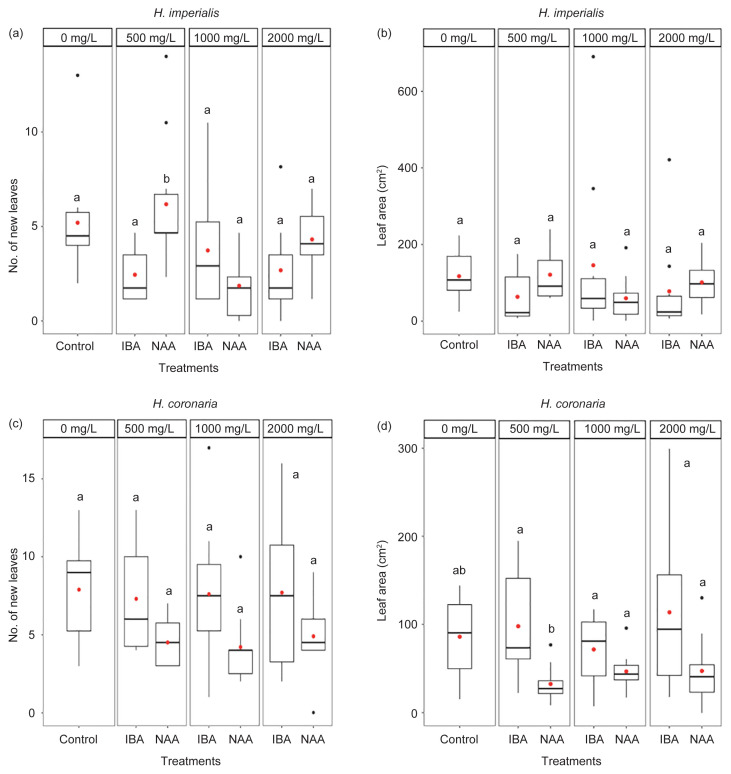
Boxplots displaying median (horizontal lines), interquartile range (boxes), maximum and minimum values (whiskers) and outliers (black dots) of number of new leaves and leaf area (cm^2^) of stem cuttings (*n* = 10 per treatment) of (a–b) *H. imperialis* and (c–d) *H. coronaria* treated with distilled water (control) and different concentrations (500, 1000 and 2000 mg/L) of hormones (IBA and NAA) after 20 weeks. Red dot within the box displays the mean value and different letters indicate significant difference in mean values as determined by Tukey’s honest significant difference (Tukey’s HSD) at 5% significance level.

Only *H. imperialis* cuttings treated with 500 mg/L NAA produced significantly more new leaves (1.2-fold increase) compared with control (*p* < 0.05; Fig. 1(a); Appendix C). In contrast, there were no significant differences in the mean numbers of leaves of *H. coronaria* cuttings between control and all hormone treatments (*p* > 0.05; Fig. 1(c); Appendix D). For mean leaf area, there were no significant differences in *H. imperialis* (Fig. 1(b); Appendix C) and *H. coronaria* (Fig. 1(d); Appendix D) cuttings between control and hormone treatments (*p* > 0.05).

#### Number of roots and length of roots

**Figure 2:**
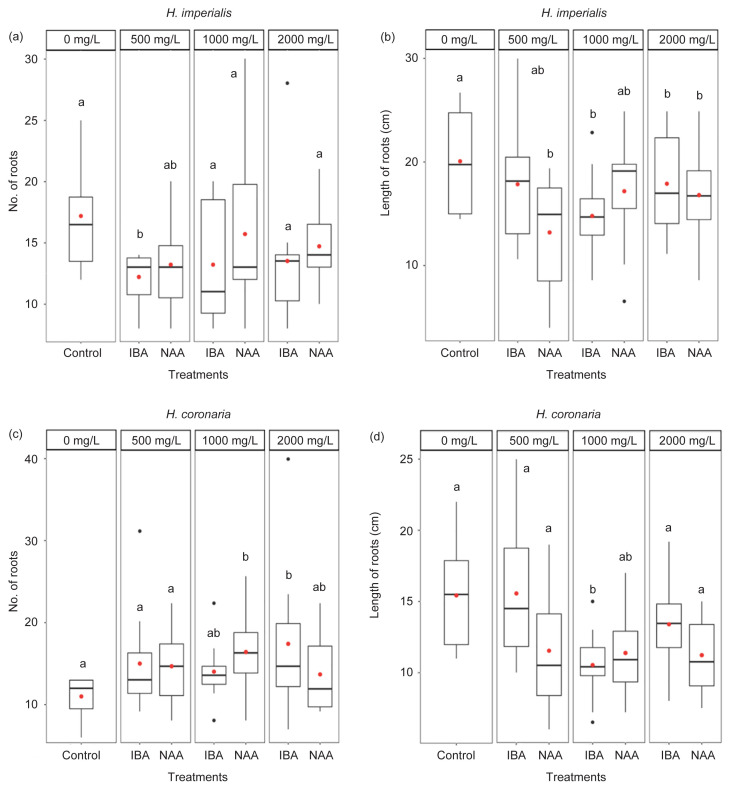
Boxplots displaying median (horizontal lines), interquartile range (boxes), maximum and minimum values (whiskers) and outliers (black dots) of number of roots and length of roots (cm) of stem cuttings (*n* = 10 per treatment) of (a–b) *H. imperialis* and (c–d) *H. coronaria* treated with distilled water (control) and different concentrations (500, 1000 and 2000 mg/L) of hormones (IBA and NAA) after 20 weeks. Red dot within the box displays the mean value and different letters indicate significant difference in mean values as determined by Tukey’s honest significant difference (Tukey’s HSD) at 5% significance level.

For *H. imperialis*, cuttings treated with 500 mg/L IBA significantly reduced the mean number of roots (1.4-fold decrease) when compared with control (*p* < 0.05; Fig. 2(a), Appendix C) but for *H. coronaria*, 1000 mg/L NAA and 2000 mg/L IBA resulted in significantly higher mean number of roots (1.5-fold and 1.6-fold increase, respectively) compared with control (*p* < 0.05; Fig. 2(c); Appendix D). *H. imperialis* cuttings treated with NAA (500 and 2000 mg/L) and IBA (1000 and 2000 mg/L) significantly reduced the mean length of roots (1.1-fold to 1.5-fold decrease) when compared with control (*p* < 0.05; Fig. 2(b); Appendix C). Similarly, *H. coronaria* cuttings treated with 1000 mg/L IBA had significantly shorter roots by a 1.5-fold reduction compared with control (*p* < 0.05; Fig. 2(d); Appendix D).

#### Relative growth rates (RGR) based on stem length and stem diameter

**Figure 3:**
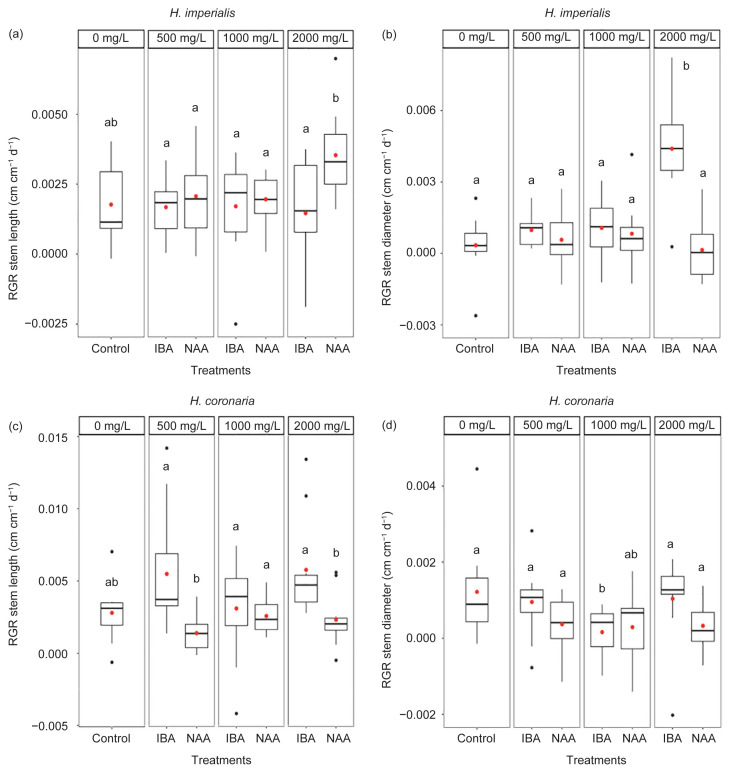
Boxplots displaying median (horizontal lines), interquartile range (boxes), maximum and minimum values (whiskers) and outliers (black dots) of relative growth rate (RGR) based on stem length and diameter (cm cm^−1^ d^−1^) of stem cuttings (*n* = 10 per treatment) (a–b) *H. imperialis* and (c–d) *H. coronaria* treated with distilled water (control) and different concentrations (500, 1000 and 2000 mg/L) of hormones (IBA and NAA) after 20 weeks. Red dot within the box displays the mean value and different letters indicate significant difference in mean values as determined by Tukey’s honest significant difference (Tukey’s HSD) at 5% significance level.

No significant differences were observed in the means of RGR based on stem length between control and hormone treatments (*p* > 0.05) for *H. imperialis* (Fig. 3(a); Appendix C) and *H. coronaria* cuttings (Fig. 3(c); Appendix D). For RGR based on stem diameter, only *H. imperialis* treated with 2000 mg/L IBA had significantly higher mean RGR based on stem diameter with an increase by 11.7-fold compared with control (*p* < 0.05; Fig. 3(b); Appendix C), whereas *H. coronaria* cuttings treated with 1000 mg/L IBA had significantly lower RGR based on stem diameter by a 7.6-fold decrease compared with control (*p* < 0.05; Fig. 3(d); Appendix D).

#### Observation of field growth

**Figure 4:**
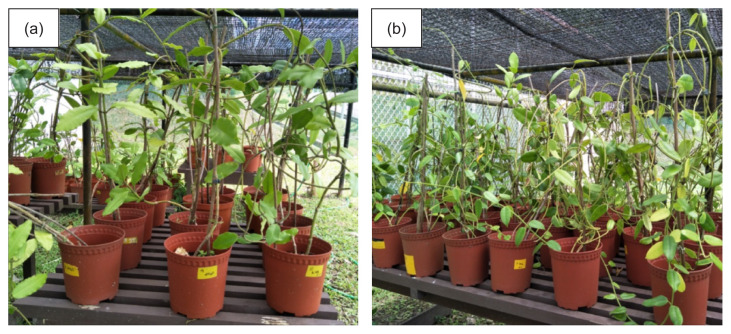
Successful propagation of (a) *H. imperialis* and (b) *H. coronaria*. The adult plants were photographed two years after transferring the plantlets from the plant house into plant shade in the field.

For each *Hoya* species, a total of 70 plantlets produced by the stem cutting method were transferred from the plant house to a plant shade in the field. No plantlets were observed to die within a one-year period of field observation (100% survival). All plantlets successfully grew to adult size, indicating that stem cutting is a viable method for propagating these species (Fig. 4).

### Callus Induction via Plant Tissue Culture

The MS media without hormone treatments or with a single hormone treatment (IBA or KN) showed no callus induction in both species (Tables 1 and 2). The leaf explants of *H. imperialis* showed callus induction in 15 out of 36 different treatments after 12 weeks (Table 1). Callus formation was observed in the media that had 0.5 mg/L IBA with 1 or 3 mg/L KN. At 1 mg/L IBA, callus was induced in the media that were supplemented with 0.5, 1 and 3 mg/L KN. Callus also developed in the media that had 3 mg/L IBA with 0.5 or 1 mg/L KN. At higher concentrations of IBA (5 mg/L and 10 mg/L), callus development was observed in the media that were supplemented with KN concentrations ranging from 0.5 to 5 mg/L. Despite displaying an overall statistically significant difference in surface areas of calluses formed among different combination treatments of IBA and KN concentrations (χ^2^(35) = 99.82; *p* < 0.001) in *H. imperialis*, none of the Dunn’s pairwise multiple comparison test showed significant differences between hormone treatments (*p* > 0.05; Table 1; Appendix A).

For *H. coronaria*, callus formation was also observed in some treatments. However, it was not as promising as observed in *H. imperialis* because only 7 out of 36 different treatments showed callus formation after 12 weeks. Callus formation was only observed when the media had 1 mg/L IBA with 0.5 or 1 mg/L KN (Table 2). The regeneration of callus was also observed in the media with 3 mg/L IBA in the presence of 0.5, 1 or 3 mg/L KN. Callus was also present in the media with 5 mg/L IBA in combination with 0.5 or 3 mg/L KN.

In *H. coronaria*, there was an overall statistically significant difference in surface areas of calluses formed among different combination treatments of IBA and KN concentrations (χ^2^(35) = 93.86; *p* < 0.001; Table 2). All the Dunn’s pairwise multiple comparison tests showed significant differences in surface areas between treatments (*p* < 0.05) except between 3 mg/L IBA with 3 mg/L KN, 1, 3 or 5 mg/L IBA with 0.5 mg/L KN, 1 or 3 mg/L IBA with 1 mg/L KN and 5 mg/L IBA with 3 mg/L KN (*p* > 0.05; Table 2; Appendix B). The mean surface area of callus appeared to be higher in *H. imperialis* than *H. coronaria*, with the highest surface area (78.4 ± 14.1 mm^2^) recorded when the medium was supplemented with 1 mg/L IBA and 0.5 mg/L KN for *H. imperialis* (Table 1; Appendix A), which was 10-fold higher than the highest surface area (7.7 ± 2.8 mm^2^) recorded for *H. coronaria* in media treated with 3.0 mg/L IBA and 3.0 mg/L KN (Table 2; Appendix B).

## DISCUSSION

The present study investigated the potential for propagating *H. imperialis* and *H. coronaria* via two different methods that utilised hormone supplementation. In stem cutting method, 500 mg/L NAA produced significantly higher mean number of leaves in *H. imperialis* stem cuttings than control. However, increasing NAA concentration to 1000 and 2000 mg/L did not further increase the number of leaves. This finding differed with the results obtained by Shahab *et al*. (2013), whereby increasing the IBA concentrations (5%–20%) increased the mean number of leaves (14.2–17.3 per cutting) in the propagation of *Alstonia* (Apocynaceae) cuttings compared with control. Similarly, another study revealed that increasing mean number of leaves was achieved in *Morus* spp. (Moraceae) cuttings with increasing concentration of NAA and that the lowest mean number of leaves was shown by control with no NAA (Pallavi *et al*. 2018).

Kumari *et al*. (2013) suggested that the increase in the number of leaves may be due to the active rooting of cuttings induced by plant hormones, which enable the roots to absorb higher amount of nutrients, and thus producing a greater number of leaves. Pallavi *et al*. (2018) also suggested that the increase in the number of new leaves may be due to greater number of roots, height of plant and branches found in each cutting. However, in the present study, it was found that 500 mg/L NAA did not significantly increase the number and length of roots in *H. imperialis* cuttings, suggesting a different mechanism for the increase in the number of leaves observed.

Stem cuttings treated with either IBA or NAA did not significantly increase the mean leaf area in both *Hoya* species. This finding is in contrast to the results obtained by previous studies (Abidin & Metali 2015; Matali *et al*. 2017) whereby IBA or NAA significantly improved the mean leaf area of *Dillenia suffruticosa* (Dilleniaceae) cuttings compared with control. The effect of IBA was also reported in *Alstonia* (Apocynaceae) cuttings, whereby the exogenous application of IBA (5%–20%) induced longer and wider leaves in comparison to control (Shahab *et al*. 2013). The results suggest that the response to different types of auxins maybe species-specific (Sevik & Guney 2013).

For *H. coronaria*, 1000 mg/L NAA or 2000 mg/L IBA treatment on the cuttings had significantly higher mean number of roots than control. However, for *H. imperialis*, the exogenous application of IBA or NAA did not significantly improve the rooting ability of the cuttings. The increase in number of roots in *H. coronaria* was probably due to the induction by the exogenous hormone (NAA or IBA) and high levels of other root-inducing substances found internally within the plant and their downward movement towards the excised ends (Rymbai & Reddy 2010; Waman *et al*. 2019). This result is consistent with those obtained by Aminah *et al*. (2006) in leafy stem cuttings of *Shorea parvifolia* (Dipterocarpaceae) whereby 8000 mg/L of IBA induced the highest percentage of rooting (63%) and Lakshmi *et al*. (2010) in *in vitro* raised shoots of *H. wightii* whereby 0.2 mg/L of IBA induced the highest percentage of rooting (90%). IBA was also previously reported to be able to initiate root formation in stem cuttings of plants belonging to the Apocynaceae family, such as *Carissa grandiflora* (Al-Mizory 2006), *Holarrhena pubescens* (Baul *et al*. 2010), *Carissa carandas* (Tanuja & Rana 2018) and *Asclepias tuberosa* (Lewis *et al*. 2020). Similarly, the exogenous application of NAA for high root formation was also reported in stem cuttings of *Vinca minor* (Zongqin & Jinxiang 1995), *Mondia whitei* (Zobolo *et al*. 2009), *Nerium oleander* (Akat *et al*. 2017) and *Nerium odorum* (Kumar *et al*. 2020), which are species from Apocynaceae.

In *H. imperialis*, the exogenous application of 2000 mg/L IBA significantly increased the mean relative growth rate based on stem diameter. According to Braun and Wyse (2019), there was an inverse relationship between stem diameter and rooting ability. It is assumed that thinner stem increases rooting ability, while thicker stem diameter reduces rooting ability. In this present study, the application of 2000 mg/L IBA produced thicker stem in *H. imperialis* and it also significantly reduced the mean length of roots but not the mean number of roots of *H. imperialis*. Thus, the increase in stem diameter seemed to have reduced the rooting ability of *H. imperialis*. However, this result differs with that obtained by Shahab *et al*. (2013), whereby high rooting ability was induced by thicker stem diameter (9.7 mm–13.2 mm) of *Alstonia* cuttings treated with 5%–20% IBA. The high number of roots per cutting was reported to increase rooting efficiency and promote nutrient uptake, thus improving the growth of cuttings.

Abidin and Metali (2015) suggested that high concentration of IBA might be toxic to cuttings and thus reducing rooting ability. The toxicity and inhibitory effect of IBA at high concentrations have also been reported in *Clerodendrum splendens* (Lamiaceae) (Jamal *et al*. 2016), *Leucadendron laxum* (Proteaceae) (Laubscher & Ndakidemi 2008) and *Duranta erecta* (Verbenaceae) (Shiri *et al*. 2019) but not in cuttings of *Holarrhena pubescens* (Apocynaceae) (Baul *et al*. 2010). On the other hand, according to Braun and Wyse (2019), a decrease in rooting efficiency in cuttings might be due to the high formation of sclerenchymatous fiber or high cortex suberization which acts as a physical barrier to the growth of adventitious roots. However, Braun and Wyse (2019) further suggested that thicker stems may have higher carbohydrate contents which can be beneficial to the cutting for maintaining its survival even without the presence of roots. Thus, the higher relative growth rate based on stem diameter of *H. imperialis* cuttings could indicate that the cuttings were more capable in storing carbohydrates needed for longer survival and also for maintaining root development.

In the callus induction experiment, callus formation was not observed when no hormone or only one hormone (IBA or KN) was present in the media for both *H. imperialis* and *H. coronaria*. Maraffa *et al*. (1980) and Rafi *et al*. (2016) similarly reported that no callus formation was observed in the explants of *H. carnosa* and *Bergenia ciliata* (Saxifragaceae) that were cultured in MS medium incorporated with 2,4-D or IBA alone, respectively. A previous study on *Catharanthus roseus* (Apocynaceae) also reported that the medium added with KN alone did not induce any callus growth (Rashmi & Trivedi 2015). These findings, however, contradicted with those that were obtained in *Mandevilla moricandiana* (Apocynaceae) (Cordeiro *et al*. 2012) whereby the incorporation of IBA alone in the MS medium was able to induce callus. Siddique (2013) also reported that the addition of 2,4-D alone in MS medium induced callus formation from the leaf segments of *H. kerrii*.

Callus formation was indeed observed when both hormones, IBA and KN were present in the media but only at specific concentrations. This is in agreement with a previous study on *Asclepias curassavica* (Apocynaceae) that showed callus developed in media supplied with combined auxin and cytokinin (Sharma *et al*. 2019). The presence of both IBA and KN at specific concentrations (generally at 0.5–10 mg/L IBA and 0.5–5 mg/L KN) was able to stimulate callus formation from the leaf explants of *H. imperialis* with the mean surface area of callus formed ranging from 0.2 mm^2^–78.4 mm^2^. Similarly, in *H. coronaria*, callus formation was also observed in the presence of both IBA and KN at specific concentrations (generally at 1–5 mg/L IBA and 0.5–3 mg/L KN) with the mean surface area of callus formed ranging from 0.4 mm^2^–7.7 mm^2^. *H. imperialis* appeared to be more suitable and promising for micropropagation compared to *H. coronaria*, as seen by the higher frequency of callus induction (i.e. 15 treatments with callus induction in *H. imperialis* compared to only 7 treatments for *H. coronaria*) as well as higher values for mean surface area of callus formed in the former. Although different plant species or varieties may respond differently to the same hormones (Kiong *et al*. 2007), other factors, such as the age of the plant, environmental condition, physiological condition of development, and nutritional state of the plant, may also contribute to the variations in hormonal response (Vimal *et al*. 2019).

Although both IBA and KN were likely required for callus formation of *Hoya* species, the concentrations of these hormones were also likely important for callus induction. The results seem to suggest that callus formation was not possible when higher concentration of KN (10 mg/L) was used in *H. imperialis*. Similarly, it appears that callus formation in *H. coronaria* was also not possible at higher concentrations of IBA (10 mg/L) and KN (5 and 10 mg/L). It was previously suggested that high concentration of KN supplied in growth media might induce toxic effects that could lead to poor plant regeneration response (Rose *et al*. 2006). A similar observation was obtained by Kiranmai *et al*. (2015) on *Caralluma pauciflora* (Apocynaceae), whereby lower concentration of IBA (3 mg/L) produced the highest induction of callus from mature internodal explants but callus induction was reduced at higher concentration of IBA (5 mg/L).

For both *H. imperialis* and *H. coronaria*, the Kruskal-Wallis non-parametric tests displayed overall significant differences in surface areas of callus formed among various combined IBA and KN treatments, however no significant differences in pairwise multiple comparison test was obtained for the former species. This finding suggests the need to conduct further study with higher replication to further determine significant pairwise differences between treatments, particularly for *H. imperialis*. For *H. coronaria*, the treatment with 3.0 mg/L IBA and 3.0 mg/L KN appeared to be the most potential treatment for callus induction as it exhibited the highest surface area compared to other treatments except for 1, 3 or 5 mg/L IBA with 0.5 mg/L KN, 1 or 3 mg/L IBA with 1 mg/L KN and 5 mg/L IBA with 3 mg/L KN. A similar result was reported by Maraffa *et al*. (1980) on *H. carnosa*, whereby callus induction was enhanced when the media were supplemented with higher concentrations of IAA or NAA than concentration of KN. However, the present result should be considered as preliminary as it needs a follow-up study with higher replication to confirm this finding.

## CONCLUSION

This study is the first to report the propagation of *H. imperialis* and *H. coronaria* using exogenous application of IBA or NAA via stem cutting method as well as the callus induction for micropropagation of both *Hoya* species using both IBA and KN via plant tissue culture. Stem cutting was found to be a more promising method for vegetative propagation of both *Hoya* species compared to micropropagation.

## Figures and Tables

**Table 1:** Mean surface area and standard error (± SE) (mm^2^) of callus formed from *H. imperialis* leaf explants after 12 weeks in media culture supplemented with different concentrations of indole-3-butyric acid (IBA) and kinetin (KN) (*n* = 4).

IBA (mg/L)	KN (mg/L)					

	0	0.5	1	3	5	10
0	0.0 ± 0.0	0.0 ± 0.0	0.0 ± 0.0	0.0 ± 0.0	0.0 ± 0.0	0.0 ± 0.0
0.5	0.0 ± 0.0	0.0 ± 0.0	22.1 ± 9.6	25.8 ± 9.7	0.0 ± 0.0	0.0 ± 0.0
1	0.0 ± 0.0	78.4 ± 14.1	0.2 ± 0.2	1.2 ± 1.2	0.0 ± 0.0	0.0 ± 0.0
3	0.0 ± 0.0	8.6 ± 2.0	4.8 ± 2.9	0.0 ± 0.0	0.0 ± 0.0	0.0 ± 0.0
5	0.0 ± 0.0	44.0 ± 15.0	21.2 ± 12.3	39.6 ± 22.9	0.4 ± 0.3	0.0 ± 0.0
10	0.0 ± 0.0	18.9 ± 6.5	8.0 ± 8.0	68.6 ± 6.7	5.5 ± 5.5	0.0 ± 0.0

*Note*: No significant differences in surface areas of callus were recorded for all pairwise comparisons between different combinations of IBA and KN concentrations as determined by the non-parametric Dunn’s pairwise multiple-comparison tests using Bonferroni’s correction (*p* > 0.05).

**Table 2:** Mean surface area and standard error (± SE) (mm^2^) of callus formed from *H. coronaria* leaf explants after 12 weeks in media culture supplemented with different concentrations of indole-3-butyric acid (IBA) and kinetin (KN) (*n* = 4).

IBA (mg/L)	KN (mg/L)					

	0	0.5	1	3	5	10
0	0.0 ± 0.0^b^	0.0 ± 0.0 b	0.0 ± 0.0 b	0.0 ± 0.0 b	0.0 ± 0.0 b	0.0 ± 0.0 b
0.5	0.0 ± 0.0 b	0.0 ± 0.0 b	0.0 ± 0.0 b	0.0 ± 0.0 b	0.0 ± 0.0 b	0.0 ± 0.0 b
1	0.0 ± 0.0 b	0.7 ± 0.5 a	1.8 ± 1,5 a	0.0 ± 0.0 b	0.0 ± 0.0 b	0.0 ± 0.0 b
3	0.0 ± 0.0 b	0.4 ± 0,4 a	6.2 ± 2.4 a	7.7 ± 2.8 a	0.0 ± 0.0 b	0.0 ± 0.0 b
5	0.0 ± 0.0 b	0.8 ± 9.4 a	0.0 ± 0.0 b	1.5 ± 1.5 a	0.0 ± 0.0 b	0.0 ± 0.0 b
10	0.0 ± 0.0 b	0.0 ± 0.0 b	0.0 ± 0.0 b	0.0 ± 0.0 b	0.0 ± 0.0 b	0.0 ± 0.0 b

*Note*:

a–b Different superscript letters show statistically significant differences in surface areas of callus between different pairs of IBA and KN concentrations as determined by the non-parametric Dunn’s pairwise multiple comparison tests using Bonferroni’s correction (*p* < 0.05).

## APPENDICES

**Appendix A:** Mean surface area and standard error (± SE) (mm^2^) of callus formed from *H. imperialis* leaf explants after 12 weeks in media culture supplemented with different concentrations (mg/L) of indole-3-butyric acid (IBA) and kinetin (KN) (Control T0; T1–T35). *n* = 4 (R1–R4).

Treatment	IBA	KN	R1	R2	R3	R4	Mean ± SE
Control, T0	0	0	0	0	0	0	0
T1	0	0.5	0	0	0	0	0
T2	0	1.0	0	0	0	0	0
T3	0	3.0	0	0	0	0	0
T4	0	5.0	0	0	0	0	0
T5	0	10.0	0	0	0	0	0
T6	0.5	0	0	0	0	0	0
T7	0.5	0.5	0	0	0	0	0
T8	0.5	1.0	32.05	43.24	13.21	0	22.12 ± 9.63
T9	0.5	3.0	23.78	45.18	34.27	0	25.81 ± 9.65
T10	0.5	5.0	0	0	0	0	0
T11	0.5	10.0	0	0	0	0	0
T12	1.0	0	0	0	0	0	0
T13	1.0	0.5	113.33	59.97	51.74	88.62	78.41 ± 14.07
T14	1.0	1.0	0	0	0.94	0	0.23 ± 0.24
T15	1.0	3.0	4.62	0	0	0	1.15 ± 1.16
T16	1.0	5.0	0	0	0	0	0
T17	1.0	10.0	0	0	0	0	0
T18	3.0	0	0	0	0	0	0
T19	3.0	0.5	13.61	7.89	9.19	3.75	8.61 ± 2.03
T20	3.0	1.0	0	7.32	11.70	0	4.75 ± 2.89
T21	3.0	3.0	0	0	0	0	0
T22	3.0	5.0	0	0	0	0	0
T23	3.0	10.0	0	0	0	0	0
T24	5.0	0	0	0	0	0	0
T25	5.0	0.5	49.85	61.12	0	65.22	44.04 ± 15.04
T26	5.0	1.0	45.03	0	39.95	0	21.24 ± 12.31
T27	5.0	3.0	75.17	0	83.22	0	39.60 ± 22.92
T28	5.0	5.0	0.99	0.75	0	0	0.43 ± 0.26
T29	5.0	10.0	0	0	0	0	0
T30	10.0	0	0	0	0	0	0
T31	10.0	0.5	21.42	24.79	0	29.72	18.91 ± 6.51
T32	10.0	1.0	0	32.05	0	0	8.01 ± 8.01
T33	10.0	3.0	86.66	70.58	60.23	56.77	68.56 ± 6.71
T34	10.0	5.0	12.46	2.76	5.72	1.04	5.50 ± 2.52
T35	10.0	10.0	0	0	0	0	0

**Appendix B:** Mean surface area and standard error (± SE) (mm^2^) of callus formed from *H. coronaria* leaf explants after 12 weeks in media culture supplemented with different concentrations (mg/L) of indole-3-butyric acid (IBA) and kinetin (KN) (Control T0; T1–T35). *n* = 4 (R1–R4).

Treatment	IBA	KN	R1	R2	R3	R4	Mean ± SE
Control, T0	0	0	0	0	0	0	0
T1	0	0.5	0	0	0	0	0
T2	0	1.0	0	0	0	0	0
T3	0	3.0	0	0	0	0	0
T4	0	5.0	0	0	0	0	0
T5	0	10.0	0	0	0	0	0
T6	0.5	0	0	0	0	0	0
T7	0.5	0.5	0	0	0	0	0
T8	0.5	1.0	0	0	0	0	0
T9	0.5	3.0	0	0	0	0	0
T10	0.5	5.0	0	0	0	0	0
T11	0.5	10.0	0	0	0	0	0
T12	1.0	0	0	0	0	0	0
T13	1.0	0.5	0	0.74	0	1.95	0.67 ± 0.46
T14	1.0	1.0 0	0.98	0	6.22	1.80 ± 1.49	
T15	1.0	3.0	0	0	0	0	0
T16	1.0	5.0	0	0	0	0	0
T17	1.0	10.0	0	0	0	0	0
T18	3.0	0	0	0	0	0	0
T19	3.0	0.5	0	0	1.77	0	0.44 ± 0.44
T20	3.0	1.0	5.06	9.45	0	10.23	6.18 ± 2.36
T21	3.0	3.0	6.26	0.97	8.73	14.63	7.65 ± 2.84
T22	3.0	5.0	0	0	0	0	0
T23	3.0	10.0	0	0	0	0	0
T24	5.0	0	0	0	0	0	0
T25	5.0	0.5	1.76	0	1.12	0.14	0.75 ± 0.42
T26	5.0	1.0	0	0	0	0	0
T27	5.0	3.0	0	0	0	6.16	1.54 ± 1.54
T28	5.0	5.0	0	0	0	0	0
T29	5.0	10.0	0	0	0	0	0
T30	10.0	0	0	0	0	0	0
T31	10.0	0.5	0	0	0	0	0
T32	10.0	1.0	0	0	0	0	0
T33	10.0	3.0	0	0	0	0	0
T34	10.0	5.0	0	0	0	0	0
T35	10.0	10.0	0	0	0	0	0

**Appendix C:** The survival percentages, rooting percentages and, mean number of new leaves, leaf area (cm^2^), number of roots, root lengths (cm), relative growth rate (RGR) based on stem length (cm cm^−1^ d^−1^) and stem diameter (cm cm^−1^ d^−1^) and standard error (± SE) of *H. Imperialis* stem cuttings treated with distilled water (control) and different types (NAA and IBA) and concentrations (500 mg/L, 1000 mg/L and 2000 mg/L) of auxins at Week 20. *n* = 10 per treatment.

Auxin treatments (mg/L)	Survival (%)	Rooting (%)	No. of new leaves	Leaf area (cm^2^)	No. of roots	Root length (cm)	RGR stem length (cm cm^−1^ d^−1^)	RGR stem diameter (cm cm^−1^ d^−1^)
Control	100	100	5.2 ± 0.95	117.28 ± 20.98	17.2 ± 1.50	20.07 ± 1.60	0.00177 ± 0.00047	0.00034 ± 0.00040
500 NAA	100	100	6.3 ± 0.96	122.71 ± 21.75	13.2 ± 1.20	13.53 ± 1.70	0.00175 ± 0.00045	0.00057 ± 0.00036
IBA	100	100	3.1 ± 0.41	65.58 ± 21.49	12.2 ± 0.66	18.10 ± 1.83	0.00138 ± 0.00032	0.00098 ± 0.00021
1000 NAA	100	100	2.6 ± 0.48	61.83 ± 18.60	15.7 ± 2.20	17.44 ± 1.66	0.00165 ± 0.00027	0.00082 ± 0.00044
IBA	100	100	4.2 ± 0.85	147.09 ± 67.48	13.2 ± 1.57	15.10 ± 1.38	0.00141 ± 0.00055	0.00107 ± 0.00040
2000 NAA	100	100	4.7 ± 0.45	102.86 ± 20.15	14.7 ± 1.01	17.07 ± 1.51	0.00314 ± 0.00049	0.00014 ± 0.00040
IBA	100	100	3.3 ± 0.65	79.77 ± 40.05	13.5 ± 1.80	18.15 ± 1.54	0.00118 ± 0.00057	0.00440 ± 0.00065

**Appendix D:** The survival percentages, rooting percentages and, mean number of new leaves, leaf area (cm^2^), number of roots, root lengths (cm), relative growth rate (RGR) based on stem length (cm cm^−1^ d^−1^) and stem diameter (cm cm^−1^ d^−1^) and standard error (± SE) of *H. coronaria* stem cuttings treated with distilled water (control) and different types (NAA and IBA) and concentrations (500 mg/L, 1000 mg/L and 2000 mg/L) of auxins at Week 20. *n* = 10 per treatment.

Auxin treatments (mg/L)	Survival (%)	Rooting (%)	No. of new leaves	Leaf area (cm^2^)	No. of roots	Root length (cm)	RGR stem length (cm cm^−1^ d^−1^)	RGR stem diameter (cm cm^−1^ d^−1^)
Control	100	100	7.9 ± 1.03	86.09 ± 14.31	11.0 ± 0.79	15.43 ± 1.17	0.00279 ± 0.000642	0.00121 ± 0.00041
500 NAA	100	100	4.5 ± 0.48	33.38 ± 6.56	15.0 ± 1.34	11.53 ± 1.30	0.00135 ± 0.000407	0.00037 ± 0.00024
IBA	100	100	7.3 ± 1.07	99.83 ± 18.97	15.3 ± 1.90	15.56 ± 1.58	0.00545 ± 0.00137	0.00095 ± 0.00031
1000 NAA	100	100	4.2 ± 0.76	47.87 ± 6.89	16.6 ± 1.38	11.37 ± 0.96	0.00254 ± 0.000387	0.00030 ± 0.00030
IBA	100	100	7.6 ± 1.42	73.12 ± 12.60	14.4 ± 1.09	10.52 ± 0.80	0.00306 ± 0.00111	0.00016 ± 0.00020
2000 NAA	100	100	48.43 ± 12.17	14.1 ± 1.35	11.22 ± 0.87	0.00228 ± 0.00060	0.00033 ± 0.00021	48.43 ± 12.17
IBA	100	100	7.7 ± 1.51	115.84 ± 29.64	17.5 ± 2.66	13.39 ± 1.05	0.00572 ± 0.00111	0.00104 ± 0.00036
